# Familial lymphoma and genetic predisposition: an updated review

**DOI:** 10.1186/s12920-026-02411-9

**Published:** 2026-06-18

**Authors:** Laura Braun, Rossella Graffeo, Kalaruban Kapilan, Harpreet Kaur Mandhair, Manuela Rabaglio, Urban Novak

**Affiliations:** 1 Department of Medical Oncology, Bern University Hospital, University of Bern, Freiburgstrasse 18, InselspitalBern, CH-3010 Switzerland; 2 Oncology Institute of Southern Switzerland, EOC, Bellinzona, Switzerland; 3 Department of BioMedical Research, University of Bern, Bern, Switzerland

**Keywords:** Malignant lymphomas, Genetic predisposition, Familial clustering

## Abstract

**Background:**

Several factors contribute to the development of malignant lymphomas including environmental exposures, bacterial and viral infections, as well as familial and genetic factors.

**Main body:**

Numerous somatic variants are involved in lymphomagenesis, and an increasing number of germline variants predisposing patients to lymphoid neoplasm have been identified. Although most lymphomas arise sporadically, epidemiological studies have linked a familial history of lymphomas and other hematological tumors with an increased lymphoma risk. Although rare, familial clustering of malignant lymphoma is well documented, and the constellations suggest inherited risk factors. Recent technological advancements have improved our ability to identify genetic factors associated with lymphoma that overall lead to a better understanding of genetic susceptibility for these entities. In this context, we here provide a comprehensive perspective on lymphoma risk factors to support improved lymphoma risk assessment and testing. A dedicated clinical management could influence future advances in lymphoma therapies and might also prevent severe toxicity and secondary malignancies. However, to date, only few susceptibility genes for malignant lymphomas have been identified. Highly penetrant mutations in known genes cannot account for most of the excess in a polygenic and heterogenic disease such as cancer. High-throughput sequencing identifies pathogenic variants that may constitute most low-penetrance alleles. However, their detection requires many well-characterized cases and controls. In this review, we therefore also include a short discussion on a Swiss cohort where we prospectively collect and samples for genetic data from individuals with a family history of lymphoma. We are convinced that such an approach is more informative and feasible than a population-based project with unselected cases and aim to better inform both genetic counsellors and oncologists.

**Conclusion:**

Inherited genetic factors contribute to lymphoma risk in a subset of patients. Improved identification of susceptibility variants, particularly through family-based studies, may enhance risk assessment and inform personalized clinical management.

## Background

Lymphoma is a cancer of the lymphatic system and can be broadly classified into two main categories: Hodgkin lymphoma (HL) and non-Hodgkin lymphoma (NHL). Within these two broad categories, there are numerous subtypes, each with its own characteristics and treatment approaches. In fact, in the current classifications of malignant lymphomas, there are > 70 different lymphoma entities [1, 2]. Both NHL and HL are among the 8 and 26 leading causes of cancer in the United States. The death rate for NHL and HL is 5.0 and 0.3 per 100 000 men and women per year respectively (SEER database, accessed August 3, 2025). Each of the specific lymphoma subtypes varies in the degree of aggressiveness, demographic distribution, risk factors, treatment strategy, and likelihood of cure [3].

Most lymphoma arise sporadically and have no known familial pathogenesis. Many factors increase the risk of lymphoma, some risk factors are common among all lymphoma, some are only associated with some entities. This suggests that there are shared as well as subtype-specific mechanisms contributing to the pathogenesis of lymphoma [4]. Epidemiological studies have linked a familial history of lymphomas and other hematologic tumors with an increased lymphoma risk [5–10]. Cerhan et al. described that a family history of hematopoietic malignancy is a replicated risk factor for lymphoma with first degree relatives of NHL, HL or chronic lymphocytic leukemia (CLL) patients having approximately an 1.7- 3.1 fold elevated risk of developing the same disease [11]. Furthermore, a large retrospective cohort study by Akthar et al. [12] from the Middle East stated that among the 1274 lymphoma patients (591 NHL, 683 HL), 10.3% reported a family member with a hematological malignancy and 9.7% had a family history of both hematological and solid malignancies.

Family history of any hematologic malignancy in first-degree relatives was found to be the most significant risk factors for NHL [4]. The Swedish family-cancer database showed that patients with a family history of NHL are at a two- to four-fold increased risk for NHL [6]. Relatives of patients with diffuse large B-cell lymphomas (DLBCL) are at a ten-fold increased risk for developing a DLBCL, a family history of follicular lymphoma (FL) increases the risk fourfold for the development of the same subtype [13]. The familial risk was shown to be stronger in females than in males and stronger for a family history of NHL in siblings than in parents (except in patients with DLBCL). However, the authors claimed that these findings might also be due to shared environmental risk factors [6]. Another study from California found that the familial risk is higher with an affected first-degree female relative than an affected male relative [14]. In addition, Cholack et al. [15] demonstrated in a cohort study of 1,994 NHL patients, that family history of hematological malignancy, present in 12.8% of cases, was associated with an inferior lymphoma-specific survival in follicular lymphoma and poorer event-free survival in mantle cell lymphoma. However, no association was observed between family history of hematological malignancy and overall survival across all NHL subtypes.

HL also harbor a firm familial component. The risk of HL in relatives of patients with HL is increased by 3.11. This familial risk is higher in males compared to females, in siblings compared to parents as well as offspring, and in families with patients with early onset of HL before the age of 40 [16].

Similar to many solid cancers, a small proportion of malignant lymphoma develop in the context of inherited susceptibility [17]. Milner et al. discovered new germline genetic syndromes that predispose individuals to lymphoma, which are associated with deregulation of the immune system [18].

Recognizing inherited predisposition to lymphoma is crucial and could potentially decrease morbidity and mortality through improved surveillance and early diagnosis in some case. This recognition also has practical implications, such as avoiding stem cell transplant from donor relatives with the same genetic predisposition, and proactive management of clinical manifestations unrelated with lymphoma such as autoimmunity, susceptibility to infection and other cancer risks [18, 19].

### Genetic predisposition

It is well known that a combination of genetic alterations and environmental factors contribute to the development of cancers including malignant lymphomas. The knowledge on environmental factors have been put together in two recent reviews [20, 21]. The individual contribution of these factors are difficult to dissect in a given individual, but also when more than one member is affected in so called “familial lymphomas”. Approximately 10% of malignancies occur in carriers of germline mutations predisposing to cancer [22]. An increasing number of identified germline might help to improve future therapy of lymphoma [23]. Germline variants in lymphoma patients are increasingly recognized as having actionable implications for therapy selection and emerging targeted treatment strategies. While the field is still evolving and no lymphoma-specific germline-directed therapies are yet FDA-approved, several lines of evidence support the potential for improved future therapy [24–27]. Lymphoma predisposition is associated with three principal mechanisms of genetic alterations: genomic instability, aberrant lymphoproliferation, immunodeficiency [19]. The genomic instability, resulting from disruption of gene involved in DNA repair, increases the general susceptibility to malignancies. Syndromes associated with an elevated risk for the development of a malignant lymphoma include Ataxia telangiectasia, Li Fraumeni; Bloom syndrome, and neurofibromatosis (Table 1). These conditions predispose individuals to multiple cancers, albeit lymphomas generally occur less frequently. Most lymphomas in a familial setting occurs in carriers with variants in genes that are not specific to lymphoma. The St Jude Lifestyle Study reported that *BRCA2* was the third most frequently mutated gene (14 occurrences) among 3006 survivors of childhood cancer, with the highest number observed among survivors of lymphoma. Further analyses of additional survivors using whole-genome sequencing showed a significant association between lymphoma, in particular NHL, and mutations in *BRCA2* [28]. Deleterious germline *BRCA1/2* variants are also frequently seen in patients with de novo hematopoietic malignancy (HM), and it is possible that they may confer risk for de novo HM, outside of the context of previous exposure to cytotoxic chemotherapy or radiation [29].

**Table 1:** Hereditary Lymphoma syndromes due to genomic instability

Syndrome	Gene	Clinical association	Clinical management	Cascade^a^	References
Ataxia telangiectasia	*ATM*	Biallelic *ATM* PV: cerebellar ataxia, telangiectasias, Immunodeficiency, pulmonary disease and risk for B cell NHL and leukemia	Biallelic *ATM* PV: neurology paediatrics, immunology, pulmonary evaluation	Recommended	76,77
(1 per 40–300,000 live births, childhood onset)					
		Monoallelic *ATM* PV (1–2% of US Caucasian adult): associated with breast, ovarian, pancreatic, prostate and colorectal cancers			
			Monoallelic *ATM* PV: screening considerations for breast, pancreatic, and prostate cancers (NCCN Clinical Practice Guidelines in Oncology (last update V3.2025)		
Li Fraumeni	*TP53*	*TP53* PV is associated with broad spectrum on cancers such as breast, soft tissue, brain, adrenal gland cancers and leukemic. Lymphoma risk is not well established	Breast, pancreatic and other cancers screening (NCCN Clinical Practice Guidelines in Oncology: (last update Version 3.2025)	Recommended	78
(1 per 3–10,000, early-onset)					
Bloom	*BLM*	Biallelic *BLM* is characterized by growth deficiency, telangiectasis immunodeficiency and predisposition to lymphoma and leukemia	Multidisciplinary assessment recommended for individuals with biallelic *BLM* PV: neurology, pediatrics, dermatology, immunology, and pulmonary specialties	Recommended	79
(rare condition, 294 individuals are known, childhood onset)			Monoallelic *BLM*: evidence insufficient to provide CRC screening recommendation, managed based on family history		
		Monoallelic *BLM* is associated low colorectal riskrisk for colorectal cancer			
Neurofibromatosis	*NF1*	Monoallelic *NF1* PV is associated with neurological disorders, glioma nerve sheath tumours and haematological cancers and childhood onset	Clinical, dermatological, neurological, and breast screening recommendations for women with *NF1*	Recommended	80,81,86
(1 per 2,000)					
Constitutional mismatch repair- MLH1 deficiency syndrome (CMMRD)	*MLH!*	Biallelic PVs cause CMMRD associated with increased risk of gastrointestinal cancers, brain and haematol. malignancies (leukemia, NHL)	The GENTURIS European Reference Network for surveillance protocols	Recommended	82,83
	*MSH2*	Monoallelic PVs cause Lynch syndrome (1 per 279) with higher risk predominantly for colorectal and endometrial cancers			
	*MSH6*				
CMMRD	*PMS2*		Colon, endometrial and other cancers screening for monoallelic PVs		
(rare disorder 200 affected individuals reported)			Colorectal, Endometrial, and Gastric (V4.2024; NCCN Network 2024 (updated May 4, 2025)		
KLHDC8B-associated familial lymphoma	*KLHDC8B*	Candidate gene for HL especially in families with a history of disease	Challenging	Challenging	62
Nijmegen breakage syndrome	*NBN*	Characterized by progressive microcephaly, growth deficiency, immunodeficiency, premature ovarian failure, and predisposition to lymphoma	Immunological, endocrine assessment and neurological evaluation	Recommended	84,85
(1 per 100,000)					

*PV* pathogenic variants, *NHL* Non Hodgkin lymphoma, *HL* Hodgkin lymphoma

^a^Genetic testing should be offered to family members as part of cascade testing, which is the process of extending genetic testing to individuals at risk of inheriting a pathogenic variant previously identified in biologic relatives [50]

Furthermore, the increasing use of tumor genetic profiling especially for relapsed or refractory lymphoma, may lead to incidental discovery of germline variants [30]. In this context a possible role of *CHEK2* and *PALB2* genes variant has been presumed [31, 32]. *Therefore*, clinical management should be considered and genetic testing for familial member is recommended (Table 1).

Genome-wide association studies (GWAS) in malignant lymphomas have identified numerous inherited susceptibility loci, particularly within immune-regulatory and HLA regions, and have substantially improved understanding of lymphoma biology [33]. However, they explain only a fraction of the heritable risk, and the "missing heritability" likely resides in rare variants with small effect sizes, gene–gene interactions, structural variants, and gene-environment interactions not captured by standard GWAS designs [34, 35]. Shi et al. have combined a transcriptome-wide association study (TWAS), a proteome-wide association study (PWAS), and a summary-data-based Mendelian randomization (SMR), to identify causal genes for NHL and HL [36].

Another mechanism contributing to lymphoma predisposition is inappropriate lymphoproliferation or stimulation (i.e., proliferation of lymphocytes or immune cells) [19]. Under normal conditions, immune cells that detect pathogenic antigens are stimulated to proliferate as a part of the immune response and as part of a highly regulated process within in germinal centers [37]. Any imbalance and deregulation between lymphocytes production and apoptosis can predispose individuals to uncontrolled cell growth and a high risk of eventual malignant transformation [37]. Syndromes associated with an increased risk of lymphoma include: autoimmune lymphoproliferative syndrome [38], and rare conditions including B cell expansion with NF- CBk к B and T cell Disease (BENTA) [23, 39], CD27 deficiency [40, 41], the Familial Hemophagocytic Lymphohistiocytosis 2 & 5 (fHLH) [42, 43], the inducible T-cell kinase deficiency [44], STAT3 gain-of-function[18], and the X-linked lymphoproliferative syndrome [45, 46]. (Table 2).

**Table 2:** Lymphoma predisposition due to inappropriate lymphoproliferation

Syndrome	Gene	Clinical association	Clinical management	Cascade^a^	References
Autoimmune lymphoproliferative	*FAS*	Defective lymphocyte regulation, autoimmunity, and predisposition for lymphoma in childhood	Challenging	Challenging	[38]
(prevalence unknown)	*FASLG*				
	*CASP10*				
B cell expansion with NF-kB and T cell Disease (BENTA)	*CARD11*	Polyclonal B cell lymphocytosis, splenomegaly and lymphadenopathy; may evolve in a B-cell lymphoma	Challenging	Challenging	[23, 39]
CD27 deficiency	*CD27*	Rare disorder characterized by lymphoproliferation and severe immune dysregulation following EBV infection	Challenging	Challenging	[40, 41]
Familial Hemophagocytic Lymphohistiocytosis (fHLH) 2 & 5	*PRF1*	Biallelic PV: excessive immune cell and cytokines activation leading to bone marrow failure. Present within the first 2 years of life with hemophagocytic lymphohistiocytosis. Unclear lymphoma risk	Multidisciplinary assessment recommended for individuals with biallelic PVs: hematology, pediatrics, immunology specialists	Challenging	[42]
1 per 100,000, childhood onset	*STXBP2*				[43]
Inducible T-cell kinase deficiency	*ITK*	Lymphoproliferation and severe immune dysregulation following EBV infection. Associated with classic HL and Hodgkin-like B-cell proliferations	Challenging	Challenging	[44]
STAT3 gain-of-function	*STAT 3*	Rare disorder characterized by lymphadenopathy autoimmune disease, infections, short stature, and possible predisposition to lymphoma	Challenging	Challenging	[18]
X-linked lymphoproliferative syndrome	*SH2D1A*	Affects males. Characterized by immunodeficiency, and severe immune dysregulation often after viral infection (e.g. EBV Severe or fatal mononucleosis, acquired dysgammaglobulinemia, hemophagocytic lymphohistiocytosis and lymphoproliferative disease in ~ 30%	Gastro-intestinal hematological, immunological and neurological assessments	Recommended	[45]
(1 per 1,000,000)	*XiAP*				[46]
Wiskott-Aldrich	*WAS*	X-linked inheritance patterns in young boys. Associated with autoimmune diseases such as autoimmune hemolytic anemia or vasculitis, risk for leukemia and lymphoma	Hematological and skin assessment	Evaluation of relatives considering stem cell donation to inform transplant donor decision	[87]
(between 1 and 10 cases per million males worldwide; rarer in females)					

*PV* pathogenic variants, *NHL* Non Hodgkin lymphoma, *HL* Hodgkin lymphoma

^a^Genetic testing should be offered to family members as part of cascade testing, which is the process of extending genetic testing to individuals at risk of inheriting a pathogenic variant previously identified in biologic relatives [50]

Immunodeficiency represents an additional factor in lymphoma risk [19]. Impairment in the immune system’s ability to recognize and eliminate non-self-substance can result in chronic, uncontrolled virus infections as well as pre-malignant or malignant cells. Disruptions in either of these pathways can elevate the risk of developing lymphoma [47]. Lymphoma predisposition related to immunodeficiencies includes conditions such as DOCK8 immunodeficiency syndrome [48] and X-linked hyper-IgM syndrome [49, 50] (Table 3). Furthermore, viruses including the Epstein-Barr virus (EBV), human T-lymphotropic virus type 1 (HTLV-1), human herpesvirus 8 (HHV-8), hepatitis C virus (HCV), hepatitis B virus (HBV), and HIV are the most well-established viral risk factors for lymphoma, each contributing to lymphomagenesis through distinct but overlapping mechanisms including direct oncogene expression, chronic antigenic stimulation, immune suppression, and epigenetic reprogramming (Table 4).

**Table 3:** Lymphoma predisposition due to immunodeficiencies

Syndrome	Gene	Clinical association	Clinical management	Cascade^a^	References
Common Variable Immunodeficiency	*TACI*	Low serum IgG, IgA, and/or IgM, loss of antibody production. Recurrent infections, autoimmune or inflammatory diseases, and increased lymphoma risk	Hematological and immunological examination	Challenging	[75]
DOCK8-immunodeficiency syndrome	*DOCK81*	Immunodeficiency and recurrent sinopulmonary and skin viral infections, elevated IgE level, atopic condition (e.g., allergies, eczema, asthma), increased risk of T-cell lymphoma, leukemia and squamous cell carcinomas	Hematological and immunological examination	Challenging	[48]
(prevalence unknown)			Curative Hematopoietic stem cell transplantation		
X-linked hyper-IgM syndrome	*CD40LG*	Affects the immune system and occurs exclusively in males. With normal to elevated IgM and low levels of the three other classes of antibodies, frequent infections in infancy, autoimmunity, inflammatory disorders, cancers of the gastrointestinal tract, and lymphoma	Hematology, immunology, pulmonary, gastrointestinal evaluation are recommended	Recommended	[49, 50]
2 per million newborn			Curative Hematopoietic stem cell transplantation		

*PV* pathogenic variants, *NHL* Non Hodgkin lymphoma, *HL* Hodgkin lymphoma

^a^Genetic testing should be offered to family members as part of cascade testing, which is the process of extending genetic testing to individuals at risk of inheriting a pathogenic variant previously identified in biologic relatives [50]

**Table 4:** Lymphoma predisposition due to viral infections

Virus	Mechanism	Lymphoma Type	Clinical Management	References
Epstein-Barr Virus (EBV)	Viral oncoproteins (LMP1, LMP2A, EBNAs) constitutively activate survival pathways (NF-κB, JNK), block B-cell differentiation, inhibit apoptosis, and enable immune evasion; establishes latency programs (I, II, III) with distinct gene expression patterns	**Burkitt lymphoma** (45.8% EBV + in HIV-negative, near-universal in endemic)	Treat lymphoma with standard curative chemoimmunotherapy	[88–90]
		**Hodgkin lymphoma** (53.0% EBV +)	No specific antiviral therapy for EBV-associated lymphomas	
		**Extranodal NK/T-cell lymphoma** (92.4% EBV +)		
		**Diffuse large B-cell lymphomas** (10.8% EBV +)	Treat lymphoma with standard curative chemoimmunotherapy (Rituximab-based)	
		**Post-transplant lymphoproliferative disorders**	Reduction of immunosuppression in post-transplant cases when feasible	
		**Primary effusion lymphoma**		
Hepatitis C Virus (HCV)	Chronic antigenic stimulation via CD81 receptor binding leads to B-cell clonal expansion, cryoglobulinemia, and progression to lymphoma through multistep lymphoproliferation; altered lymphocyte homeostasis	**Marginal zone lymphoma** (esp. splenic MZL)	**Indolent lymphoma (MZL):**	[91, 92]
		**Diffuse large -cell lymphomas** (often arising from transformed MZL)	Direct-acting antivirals (DAAs) as first-line therapy; achieves lymphoma response in ~ 45–73%	
		**Lymphoplasmacytic lymphoma**	Sofosbuvir-based regimens for 8–12 weeks Sustained virologic response > 95%	
			**Aggressive lymphoma (DLBCL):**	
			Standard R-CHOP first	
			DAAs concurrent with or after chemotherapy to reduce hepatotoxicity	
			Monitor liver function and HCV RNA during treatment	
Human T-cell Lymphotropic Virus Type I (HTLV-1)	Viral oncoproteins Tax and HBZ modulate signaling pathways, evade host immunity, and drive T-cell transformation; requires prolonged infection with accumulation of mutations before malignant transformation	**Adult T-cell leukemia/lymphoma (ATL):**	**Smoldering/favorable chronic:**	[93, 94]
		Acute subtype	Watchful waiting or interferon-α/zidovudine	
		Lymphoma subtype	**Unfavorable chronic/acute/lymphoma:**	
		Chronic subtype (favorable and unfavorable)	Preferred: CHOP + mogamulizumab, dose-adjusted EPOCH, or interferon-α/zidovudine	
		Smoldering subtype	CNS prophylaxis recommended	
			Allogeneic HSCT for eligible patients	
			**Relapsed/refractory:**	
			Mogamulizumab, lenalidomide, or brentuximab vedotin (CD30 +)	
			Salvage chemotherapy	
Human Herpesvirus 8 (HHV-8/KSHV)	Viral proteins activate NF-κB, JAK/STAT, and PI3K/AKT pathways; produces viral IL-6 driving inflammatory cytokine dysregulation; enables immune escape; often co-infected with EBV (~ 80%)	**Primary effusion lymphoma (PEL)**	*PEL/HHV-8* + *DLBCL:*	[95, 96]
		**HHV-8 + DLBCL NOS**	Preferred: EPOCH (etoposide, prednisone, vincristine, cyclophosphamide, doxorubicin) with antiretroviral therapy (ART) in HIV +	
		**Multicentric Castleman disease**	Consider rituximab + EPOCH if concurrent MCD	
			Rituximab not indicated if CD20-negative	
			G-CSF support for all patients	
			*Emerging therapies:*	
			Lenalidomide + rituximab combinations	
			Bortezomib-based regimens	
			Daratumumab under investigation	
			Siltuximab/tocilicumab for idiopathic MCD only	
Hepatitis B Virus (HBV)	HBV X protein inhibits p53 tumor suppressor; chronic inflammation and immune dysregulation; direct and indirect oncogenic mechanisms including chromosomal translocations and antiapoptotic gene activation	Non-Hodgkin lymphoma (contributes to ~ 8% of cases):	Screen all patients with HBsAg and HBcAb before anti-CD20 therapy	[97]
		**Diffuse large B-cell lymphomas**	**HBsAg-positive:** Prophylactic entecavir (preferred) during and for 12 months after anti-lymphoma therapy	
		**other B-cell lymphomas**	**HBcAb-positive:** Prophylactic entecavir preferred; monitor if high-level HBsAb present	
			Monitor HBV viral load monthly during treatment, every 3 months after	
			Consult hepatology if viral load increases	
			Treat lymphoma per standard protocols	
Human Immunodeficiency Virus (HIV)	Profound immunosuppression (when HIV infection is unknown) creates permissive environment for oncogenic viruses (especially EBV, HHV-8); chronic immune activation and dysregulation	High-grade non-Hodgkin lymphoma:	Concurrent antiretroviral therapy (ART) with chemotherapy strongly recommended; associated with higher CR rates	[98]
		**Diffuse large B-cell lymphomas**	Avoid zidovudine, cobicistat, ritonavir during chemotherapy	
		**Burkitt lymphoma**	Standard lymphoma-directed therapy per subtype	
		**Primary effusion lymphoma**	G-CSF support	
		**Plasmablastic lymphoma**	Maximize supportive care if CD4 50	
		**HHV-8 + DLBCL**	CAR T-cell therapy (to treat a relapse) appropriate with HIV control measures	
			Consult HIV specialist for ART optimization	

### Familial non-Hodgkin lymphoma

Although most of lymphoma cases arise sporadically, around 5% are caused by familial predisposition in combination with environmental factors [51]. According to Houlston & Peto [52], studies analysing patients with familial aggregation of cancer are more informative than those including unrelated cancer patients. This might also explain why certain predisposing mutations to lymphoma could not be replicated nor have they been studied in the wet lab and therefore remain questionable. To our knowledge, there are so far only a very limited number of studies that performed exome sequencing on families with familial aggregation of NHL.

Saarinen et al. [53] performed exome sequencing on a Finnish family with three siblings with a primary mediastinal B cell lymphoma (PMBCL) and their cousin with an extranodal DLBCL. The only variant found in all four patients was a mutation in the *MLL* gene, which suggests a contribution to the familial predisposition of PMBCL. This variant was not found in healthy controls outside the family, but in healthy family members.

Chan et al. [54] reported two male siblings from a non-consanguineous Chinese family who both developed extranodal NK/T-cell lymphoma (NKTL), a rare lymphoma entity. Whole-exome sequencing of the affected brothers and unaffected relatives identified a homozygous missense germline mutation in FAM160A1. The unaffected mother was a heterozygous carrier, while the unaffected older brother was homozygous wild-type. FAM160A1 is known to interact with a protein complex involved in vesicle trafficking. These findings suggest that a recessive germline mutation in FAM160A1 may represent a potential genetic predisposition factor for familial NKTL.

Wang et al. [25] performed exome sequencing on 18 lymphoma patients from 13 lymphoma-cancer families, including 27 lymphoma and 26 other cancer cases. The study demonstrated an enrichment of protein-truncating variants (PTVs) in DNA repair genes (ATM and PNKP) and 14 immune genes. PTVs in ATM and PNKP were each identified in two families.

We recently studied a Swiss/Japanese family [55] where one sister was affected by PMBCL and another by a mediastinal non-germinal centre DLBCL. Whole-exome sequencing was performed on the germline DNA of the two affected sisters and the core family: a variant in the *TIRAP* gene was identified in both sisters and their Japanese mother but was absent in other healthy family members. Subsequent functional experiments revealed that the variant enhanced the activity of the NF- кB signalling and reduced cell death, thus likely contributing to lymphomagenesis in this pedigree.

### Familial Hodgkin lymphoma

A strong familial aggregation of HL has been described [56] and several studies investigated families with multiple HL patients: Lawrie et al. [57] performed whole-exome sequencing on the germline DNA of a family in which five family members were diagnosed with classical HL. They identified alterations in two genes that might increase susceptibility to familial lymphoma: *FAM107A,* a tumour suppressor gene, and *SKC26A6*, coding for an anion carrier whose role in lymphomagenesis is unclear.

Sixty five families with two or more HL patients were analysed by Rotunno et al. [56]. The same mutation in the *KDR* gene, coding for a type III receptor tyrosine kinase with an impact on proliferation, migration and survival, was found in two of these 65 families. A mutation in the “Protection of Telomeres 1” gene, which plays a role in telomere maintenance, was found in two of 41 families analysed by McMaster et al. [58]. In a Finnish family with four cousins affected by nodular lymphocyte predominant HL, Saarinen et al. [59] found a mutation in the so-called “nuclear protein, ataxia-telangiectasia locus (NPAT)”. All cousins and three of five healthy family members carry this variant, which suggests a low penetrance or involvement of additional genetic and environmental factors. Bandapalli et al. [60] analysed germline DNA of two family members affected by cHL as well as one unaffected family member and found a variant of DICER1. This germline mutation was only present in affected family members, suggesting a possible contribution of *DICER1* in the development of cHL through an impaired expression of the tumour-suppressing miRNA. Srivastava et al. [61] validated this *DICER1* variant as a possible predisposing factor for cHL in a German family. Furthermore, a depletion of KLDHC8B was found by Salipante et al. [62] in a family with multiple cases of cHL. This reduced expression leads to the binucleation of cells, which is a characteristic of Reed/Sternberg tumour cells. In a family with three cHL patients, a homozygous deletion in the *ACAN* gene was found in all patients by Ristolainen et al. [63]. The healthy family members displayed both a heterozygous and wild-type status of this gene.

In a larger cohort, Flerlage et al. [64] performed whole genome sequencing on 234 individuals from 36 pedigrees with two or more first-degree relatives with HL. Recurrent variants included coding mutations in KDR and KLHDC8B, as well as noncoding intronic variants in PAX5 and GATA3. In addition, several novel candidate genes were identified, including IRF7, EEF2KMT, and POLR1E.

### Whole-exome sequencing and pedigree analysis

Whole-exome sequencing covers over 95% of the exons, the protein-coding regions of the genome, enabling the detection of genetic alterations [65]. There are two types of exome sequencing (ES): Whereas singleton-ES is performed on the proband only, trio-ES uses additional samples including both biological parents. In a head-to-head comparison, tES did not show a higher molecular diagnostic rate than sES [66]. Furthermore, tES is almost twice as expensive as sES when considering analysis and sequencing [66]. However, Lee et al. claimed superiority of tES compared to sES due to a higher sensitivity to detect de novo and compound heterozygous variants [67]. To our knowledge, little is known about which individuals of a pedigree whole-exome sequencing needs to be performed to achieve a high-sensitivity detection of genetic disorders in familial lymphoma cases. In the Finnish study from Saarinen et al. [53] discussed earlier, exome sequencing was performed to determine genetic disorders in a family with three siblings with PMBCL and a cousin with an extranodal DLBCL. Blood-derived DNA was available from all patients and from four healthy family members: one parent, two kids and one healthy sibling. There was no DNA analysed from close family members of the affected cousin [53]. Also in the other study by Saarinen et al. [59] tumour tissue DNA of lymphoma patients and blood-derived DNA of healthy donors as a control were used. The authors did not specify why they did not include more family members in the analysis, but likely, this was not available.

Recent sequencing studies have provided further insight into the genetic basis of familial lymphoma. In a study published in 2026, Ralli et al. [68] performed exome sequencing in 82 affected and 12 unaffected individuals from 43 families with at least two cases of lymphoid cancer. They used the “Weights-based vAriant Ranking in Pedigrees (WARP)” pipeline, a variant prioritization framework developed for identifying susceptibility variants in complex familial disorders. This is especially useful when analyzing a mixture of small and large pedigrees with possible genetic heterogeneity. Six affected unrelated probands were used for the interpretation only. The most frequently recurrent germline variant was found in 9% of families in the gene FAM160A1. In addition, variants in genes of the WNT/β-catenin signalling pathway (BCL9, LEF1, TLE3 and KLHL12), NPAT, HCLS1 and ID3 were identified. While some variants segregated with specific lymphoma subtypes, others were shared across different subtypes. These findings build on an earlier work by Ralli et al. [35], in which burden analyses on a subset of 39 families (one case per family) identified four additional germline genes, including INTU, PEX7, EHHADH, and ASXL1, and highlighted pathways involved in innate and adaptive immune systems and peroxisomal metabolism.

In a larger case–control study by Usui et al. [24], who analysed 27 cancer-predisposing genes in 1,982 Japanese lymphoma patients and 37,592 controls, significant associations between pathogenic germline variants in ATM (OR 2.63), BRCA1 (OR 5.88), BRCA2 (OR 2.94), and TP53 (OR 5.22) and lymphoma risk were identified, with a particularly strong association for mantle cell lymphoma (OR 21.57). Overall, 1.6% of lymphoma patients carried pathogenic variants in these genes.

### Familial lymphoma cases in Switzerland

Familial lymphomas account for around 5% of all lymphoma cases. First-degree relatives have an increased risk to also develop a for lymphoma. These findings indicate a genetic component to lymphomagenesis. There are many known somatic mutations known that contribute to lymphomagenesis [69, 70], but our knowledge on molecular characteristics of familial lymphoma cases is, as discussed earlier, currently limited to few pedigrees.

**Fig. 1:**
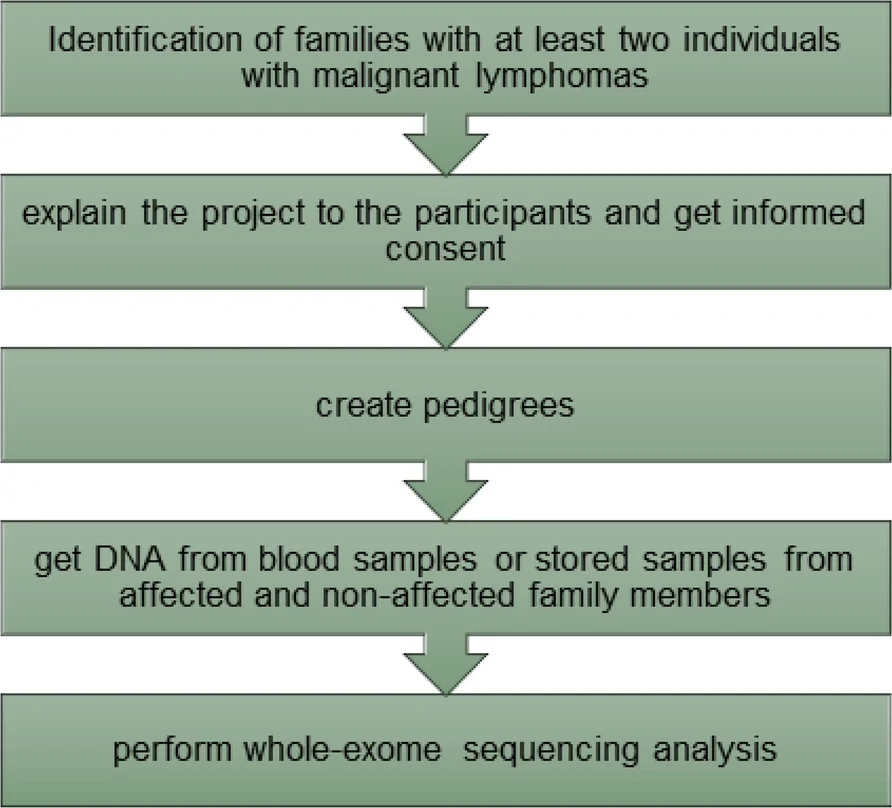
Outline of a project to assess familial lymphoma cases. The title of the ongoing project is "Whole-exome sequencing analysis of familial lymphoma cases in Switzerland—a multicentric research project"

We recently initiated a multicenter project called “*Whole-exome sequencing analysis of familial lymphoma cases*” in Switzerland (Fig. 1). This translational project aims to improve understanding of familial predisposition to lymphoma cases by discovering underlying genetic alterations in genes possibly contributing to lymphomagenesis. To identify familial lymphoma cases in Switzerland, we conducted a survey among major haemato-oncologists representing both university and larger regional hospitals. With the ethical approval (KEK Bern 2020–01110), we started to contact the families and to draw the pedigrees of the families that agreed to participate (outline provided in Fig. 1). We aim to collect blood-derived or stored samples of all available affected and non-affected family members and to perform whole-exome sequencing analysis on the DNA. We so far collected six complete pedigrees (*data not shown*).

**Fig. 2:**
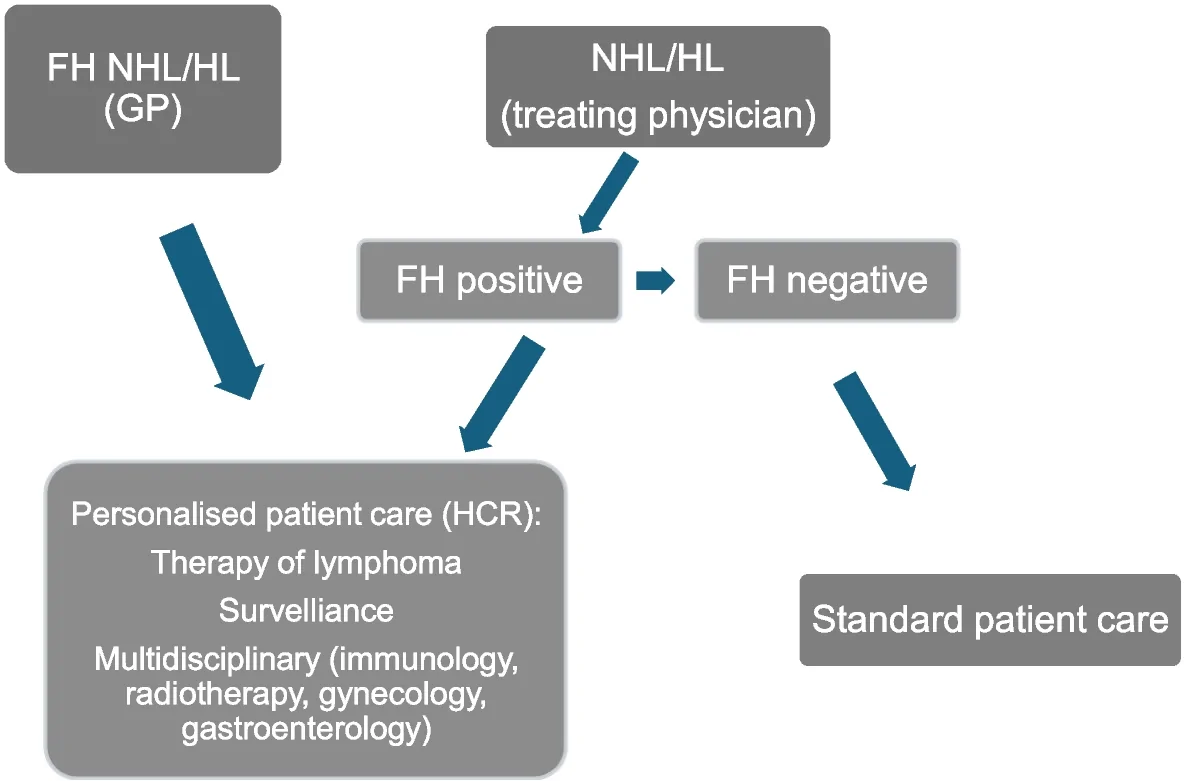
Identification of genetic predisposition to lymphoma FH: family history, GP: general practitioner, HRC: High risk clinic

In Switzerland, genetic studies are amenable to the “Federal Act on Human Genetic Testing (HGTA)” [Available from: https://www.fedlex.admin.ch/eli/cc/2007/131/en], ratified by the Federal Assembly of the Swiss Confederation. As mentioned in article 5 of the HGTA, the participants must be “provided with adequate information” to be able to give or refuse consent. Therefore, we had to create comprehensive information sheets for all possible participant groups. We developed individual documents for adults, adolescents aged 14 to 17 years, children up to 14 years, parents, legal representatives and relatives. This is important given the possibility that as part of this pure research project, a potentially harmful known germline alteration could be discovered by change. This might trigger preventive or therapeutic measures that not a priori foreseen in this project. In national and international guidelines, the right not to know requires an explicit request [UNESCO: Universal Declaration on the Human Genome and Human Rights 1997, available from: https://www.unesco.org/en/legal-affairs/universal-declaration-human-genome-and-human-rights; World Medical Assembly (WMA) Declaration of Lisbon on the Rights of the Patient 1981, revised 2005, available from: https://www.wma.net/wp-content/uploads/2005/09/Declaration-of-Lisbon-2005.pdf] [71]. Importantly, so far, all participants in our ongoing project who decided to participate agreed to be informed about clinically relevant findings. However, in addition, any subsequent actions will also follow the principle of informed consent and the right of self-determination (Art. 3 WMA). At this point, we cannot yet report on the molecular analysis of the samples. Due to the expected low-penetrance and the heterogeneity of malignant lymphomas, it would be a surprise to find the same variant in more than one family. Studies on malignant lymphomas such as DLBCL have so far identified potentially targetable *somatic* mutations to improve treatment and have yielded a wealth of information on the genetic landscape [69, 70]. This prospective project may complement our understanding *of* malignant lymphomas and ideally identify new therapeutic targets. The identification of an inherited TIPAP variant [55] supported the important role of TLRs/NF- κB in the pathogenesis in DLBCL and provides a rational for the use of novel agents such as IRAK4 inhibitors [72]. In Fig. 2, we propose how to identify a possible genetic predisposition to lymphoma with the current knowledge.

## Conclusion

This review offers a comprehensive overview of the major mechanisms contributing to lymphoma predisposition including genomic instability syndromes, immunodeficiencies, and aberrant lymphoproliferations as well as virus infections. The clinical application of this knowledge is supported by recommendations for effective collection of personal and family medical history to inform risk assessment, lymphoma surveillance and testing. The study of familial lymphoma and genetic predisposition is hampered by the absence of dedicated international registries, under ascertainment of family history, technical barriers to germline testing in liquid tumors and diversity gaps, placing lymphoma far behind solid tumors in the hereditary cancer landscape. The prospective multicenter project in Switzerland was started to address this gap by identifying families with multiple lymphoma cases. The expanded use of PARP inhibitors on cancers other than breast or ovarian cancer [73] and the detected significant activity of checkpoint-inhibitors in microsatellite instability-high cancers [74, 75] led to an increased demand on genetic counselling. Very few such therapeutic options currently exist in the vast therapeutic armamentarium of lymphomas, however, this might change based on recent results [24–27]. In addition, lymphoma is not traditionally associated with inherited cancer syndromes, but patients, not only from families with more than one affected member are increasingly asking for such information.

## Data Availability

No datasets were generated or analysed during the current study.
